# Parental concern for clinically vulnerable child during first 18 months of the COVID pandemic

**DOI:** 10.1038/s41390-022-02371-7

**Published:** 2022-11-22

**Authors:** Corine Driessens, Lynne Mills, David Culliford, Ravin Patel, Emma Lee, Diane Gbesemete, Harry Chappell, Meera Shaunak, Saul N. Faust, Hans de Graaf, On behalf of the ImmunoCOVID19 study group (Appendix A)

**Affiliations:** 1 NIHR Applied Research Collaboration Wessex, University of Southampton, Southampton, UK; 2 NIHR Southampton Clinical Research Facility and Biomedical Research Centre, University Hospital Southampton NHS Foundation Trust Mailpoint 218, University Hospital Southampton NHS Foundation Trust, Southampton, UK; 3 Faculty of Medicine and Institute for Life Sciences, University of Southampton, Southampton, UK

## Abstract

**Background:**

The uncertainties surrounding the COVID-19 pandemic have been associated with increased parental concern. The aim of this study is to explore if this increased level of concern is associated with certain individual/household characteristics or if parents adapted to the ever-changing realities of the COVID-19 pandemic over time.

**Methods:**

This prospective study explored COVID-19 concern trajectories and associated family characteristics of 765 UK parents caring for an immunosuppressed child during the first 18 months of the pandemic using growth mixture modelling. Qualitative analysis was performed to examine in more detail the source of concern.

**Results:**

Four different trajectories of parental COVID-19 concern were identified. Ongoing very high concern was associated with caring for children with nephrotic or respiratory disease; having a child on an organ transplant waiting list; residency in the North of England; or parental vocational inactivity. Explicit concerns voiced by the parents generally followed national trends, but vulnerable status specific concerns were also reported.

**Conclusion:**

Diagnosis and prescribed medication of the immunosuppressed child, geographical location, household composition, and employment status of parent were associated with the different concern trajectories. This information can be helpful in targeting psychological family care where it is most needed.

**Impact:**

- Many British parents caring for a clinically vulnerable child during the first 18 months of the COVID-19 pandemic showed high levels of concern with little sign of psychological adaptation.
- Consistent with findings from non-vulnerable populations, parents mentioned the impact of shielding and repeated isolation on their child’s education, social life, and mental health.
- Unique to the clinically vulnerable population, parents were worried about child’s health status, impact of delayed healthcare, and were confused by the contradictory information received from government, doctors, and media.
- Psychological family care can be targeted to those parents at greater risk for high levels of concern.

## Introduction

In March 2020 United Kingdom (UK) residents believed to be at high risk for severe illness from SARS-CoV-2 infection were advised to “shield”. For immunosuppressed children this initial period of family isolation lasted until July 2020 when the strict guidance for those deemed clinically extremely vulnerable (CEV) to SARS-CoV-2 was gradually relaxed. As the pandemic progressed, children were shown to be less affected by SARS-CoV-2 than adults^1^ and most immunosuppressed children were removed from the CEV list, despite their generally high risk for other bacterial and/or viral infections^2^. UK children, including most immunosuppressed children, returned to school in September 2020, and continued to attend during the second British lockdown in November 2020. After the 2021 Christmas Holiday period a third lockdown came into effect in the UK which included a closure of the schools. During this period, immunosuppressed children were home-schooled alongside their peers. In March 2021 the British government introduced a four-step roadmap out of lockdown^3^, which included the opening of schools on March 8^th^ 2021. With the slow relaxation of social restrictions and the proportionate case rise of the delta SARS-CoV-2 variant in the UK, the number of COVID-19 cases started rising again, especially among the younger generation^4^. Despite the ongoing UK transmission of SARS-CoV-2, the last step of the roadmap out of lockdown was initiated on July 19th 2021. Once all British children aged 16/17 had been offered the COVID-19 vaccine (August 2021), children aged 12 to 15 were scheduled for vaccinations (September to December 2021) followed by children aged 5 to 12 (April 2022 onwards). Still the autumn and winter of 2021/2 was marked by high school-absence rates among children as well as teachers due to COVID-19-related reasons. Some limited COVID-19 restrictions, known as the plan B measures, were implemented for January 2022 and in April 2022 England moved into the ‘Living with COVID-19’ phase. Overall, compared to pre-COVID-19, the number of children being home-schooled in England has risen. A few immunosuppressed children remained on the CEV list throughout COVID-19 pandemic and continued to shield, often due to conflicting advice provided from central NHS messaging compared to local or specialist clinicians. However, in contrast to the initial pandemic months, family members were required to return to work or school on August 1^st^ 2020.

The ever-changing COVID-19 guidelines and restrictions drastically altered everyday life. In the vulnerable population, the isolation, modified routine, and fear of exposure experienced during the COVID-19 pandemic could have intensified the challenges already experienced by families living with a vulnerable child. In many adults the isolation and uncertainty caused by the first UK lockdown triggered mental health problems, including anxiety^5,6^. As adults adapted to this disruptive life event, some reported that mental health problems diminished^7^, yet parents and especially mothers continued to be at higher risk for emotional mental health problems^8^. Data from the second lockdown shows that the depressive symptoms and stress experienced by UK parents increased to even higher levels compared to the first lockdown^9^.

Research conducted amongst parents/carers of clinically vulnerable children suggested that parents/carers of children with chronic illness experienced an increase of emotional mental health problems, stress, and worry during the COVID-19 pandemic^10,11^. However, these studies involved relatively small cohorts and focused on only a few discrete timepoints during the pandemic. To gain further insight into the psychological wellbeing of parents caring for vulnerable children during the first 18 months of the pandemic, we explored the weekly data collected by the ImmunoCOVID-19 study^12,13^. The data from this study allowed us to explore both the levels of concern expressed by parents caring for an immunosuppressed child during the first 18 months of the COVID-19 pandemic in the UK and the characteristics associated with different parental concern trajectories. It also allowed us to examine how the content of parental concerns changed before, during, and after the easing of the three lockdowns in the UK.

## Methods

### Participants

ImmunoCOVID-19 is a large-scale prospective study involving weekly surveys which capture daily clinical and life experience data for more than 1600 immunocompromised paediatric patients recruited from 46 hospitals across the UK. In accordance with the UK health guidelines, immunocompromised was defined as having a medical indication for an annual influenza vaccine. Ethical approval for this study was received from Leeds NHS Research Ethics Committee (IRAS 281544). The analysis described here focused on the experiences of the parents of these patients under 18 years of age. 2856 parents of immunocompromised children were referred to the study by their child’s clinical team as they expressed interest in participation. Those who completed an online consent form (*n* = 1631/2856, 57.1%), were sent weekly online questionnaires to complete. We focused on the parents of 765 (46.9%) patients who completed at least one of the weekly questionnaires in each of the following periods: the shielding period (March 16th to July 31st 2020), the period of relaxed restrictions for vulnerable individuals (August 1st 2020 to January 4th 2021), the third UK lockdown (January 5th to March 7th 2021), and the roadmap out of lockdown (March 8th to September 26th 2021).

### Measures

The weekly ImmunoCOVID-19 online survey is described in detail elsewhere^12^. In summary, the survey collects data regarding the immunosuppressed child’s diagnoses and medications, weekly symptom presentation, COVID-19 testing and results, COVID-19 vaccinations, NHS attendance, and hospital admissions. SARS-CoV-2 infection is first determined by self-report on the weekly survey and then verified via personal email to make sure diagnosis was obtain from PCR testing. In addition, the survey records if the child has been missing out on school (yes/no) and/or sport/leisure activities (yes/no). In this study, the effects on school, sport/leisure have been used as an approximation of the pandemic’s effect on the child’s daily life. Furthermore, parental concern was assessed by a quantitative variable (“On a scale of 0 [not worried] to 10 [extremely worried], how worried are you about coronavirus affecting your child?”) and a qualitative variable (“Is there anything that you are particularly worried about that you would like to share?”). Demographic information (gender and age of parent and child) as well as household characteristics (geographical location, rural/urban, green space, household income, household composition, and employment parent) were also collected.

### Quantitative analysis

Latent class growth analysis (LCGA) was employed to identify the multiple distinct patterns of change in parental concern present in the first 18 months of the COVID-19 pandemic^14,15^. This modelling cannot be done within the base SAS9.4 package and so the PROC TRAJ extension was deployed (http://www.andrew.cmu.edu/user/bjones). The data was restructured into a wide format and categorical covariates were changed into dummy variables to adhere to the requirements of the PROC TRAJ guidelines.

The outcome variable ‘parental concern’ was measured on a continuous scale from 0 to 10 assuming a normal distribution. Censored normal PROC TRAJ models were used to represent the patterns of concern over time. This type of group-based modelling assumes that a finite number of different patterns of concern can be found among the data. To determine the number of patterns that best fit the data we compared different concern pattern models. First, we fitted a one-pattern model, then a 2-pattern model, and so on until a 5-pattern model was fitted. Following advice from Nagin^16^ regarding this first phase of the analysis, the polynomial order (shape) of the trajectories were set to quadratic. To decide on the optimum number of distinct parental concern patterns for our data the nested models were compared on Bayesian Information Criterion (BIC) and Akaike Information Criterion (AIC); the best fit model is the one with the smallest negative number. In addition, average posterior probabilities were calculated to indicate the average probability that individuals grouped in the same trajectory belong to the same class given their observed trajectory of concern over the 18 months of the pandemic. Models with an average posterior probability >0.7 for each group indicated better accuracy of pattern membership. Finally, we adhered to Nagin’s^16^ recommendation of group size of at least 5% of the sample size and above.

Once the number of distinct patterns was identified, we went to the second phase of the analysis and determined the best shape (straight line, linear increase or decrease over time, quadratic pathway over time, cubic pathway over time) for each distinct parental concern pattern. Within the PROC TRAJ command we adjusted the polynomial order for each trajectory until the highest order polynomial term for each distinct parental concern pattern was significant at a two-sided 5% level.

Once the optimum shape and number of parental concern patterns was chosen, differences between the patterns were explored by determining the association of time-stable covariates with the different growth patterns. The predictive ability of a time-stable covariate was calculated by a multinomial logit function estimating likelihood of membership to a distinct parental concern pattern based on the level of the time-stable covariate. The ‘medium concern—full adaptation’ group was chosen as the reference group.

Not all parents disclosed time-stable covariates (e.g. demographic characteristics, household facts, diagnostic information or medication use details) for their immunosuppressed child, thereby limiting the sample size for these analyses.

### Qualitative analysis

The transcripts were analysed using nVivo 12.0^17^. A reflexive thematic analysis was adopted^18^. Themes were identified inductively, similar sub-themes then grouped together under an overarching thematic framework.

## Results

Our analytic sample consisted of 765 parents of immunosuppressed children who participated in 12 to 78 of our weekly surveys, providing in total of 38,449 observations with a median of 52 observations per parent. Household characteristics for these parents are provided in the ‘Full sample’ column of Table 1a and characteristics for their immunosuppressed child are provided in ‘Full sample column’ of Table 1b.

**Table 1:** a Household characteristics. b Characteristics of immunosuppressed children

**a**							
	**Full sample**	**Differential concern groups**				**Chi-square**	***p***
		**Medium concern, full adaptation**	**Medium concern, some adaptation**	**High concern, some adaptation**	**High concern, no adaptation**		
*Gender parent* female *N* (%)	335 (89%)	60 (86%)	111 (87%)	122 (90%)	42 (95%)	5.02	0.54
*Age parent* median(range)	44 (27–62)	45 (27–59)	44 (30–58)	45 (27–62)	42 (32–56)	4.46**	0.22
*Employment parent* *N* (%)						21.65	0.001
Fulltime	135 (36%)	26 (38%)	46 (37%)	53 (39%)	10 (23%)		
Parttime	159 (43%)	32 (47%)	62 (50%)	50 (37%)	15 (35%)		
Not working, disabled, retired	75 (20%)	10 (15%)	15 (12%)	32 (24%)	18 (42%)		
*Household composition* *N* (%)						12.64	0.05
Single parent w/child(ren)	51 (14%)	17 (24%)	13 (11%)	12 (9%)	9 (20%)		
Couple w/child(ren)	298 (80%)	48 (69%)	103 (84%)	115 (85%)	32 (73%)		
Couple w/child(ren) w/relatives	23 (6%)	5 (7%)	7 (6%)	8 (6%)	3 (7%)		
*Number of children* median (range)	2 (1–12)	2 (1–5)	2 (1–5)	2 (1–12)	2 (1–3)	0.58	0.90
*Household income* *N* (%)						9.45	0.15
>£29,500	236 (64%)	44 (65%)	85 (69%)	86 (64%)	21 (49%)		
~£29,500	64 (17%)	9 (13%)	23 (19%)	23 (17%)	9 (21%)		
<£29,500	69 (19%)	15 (22%)	15 (12%)	26 (19%)	13 (30%)		
*Geography N* (%)							
Scotland	51 (13%)	14 (19%)	16 (13%)	16 (12%)	5 (11%)	2.83	0.42
Wales	15 (4%)	4 (6%)	2 (2%)	7 (5%)	2 (4%)	2.99*	0.31
North England	90 (24%)	12 (17%)	30 (23%)	30 (22%)	18 (40%)	8.75	0.03
Middle England	65 (17%)	12 (17%)	21 (16%)	24 (18%)	8 (18%)	0.11	0.99
South England	159 (42%)	30 (42%)	59 (46%)	58 (43%)	12 (27%)	5.28	0.15
*Urbanisation* *N* (%)						6.80	0.34
<2500 habitants	49 (13%	10 (14%)	20 (16%)	17 (13%)	2 (5%)		
2501–10,000 habitants	123 (33%)	17 (24%)	42 (33%)	46 (34%)	18 (41%)		
>10,000 habitants	202 (54%)	43 (61%)	64 (51%)	71 (53%)	23 (55%)		
*Access to green space N* (%)						7.15	0.07
Easy	354 (94%)	67 (96%)	124 (97%)	125 (93%)	38 (86%)		
(Somewhat) Difficult	23 (6%)	3 (4%)	4 (3%)	10 (7%)	6 (14%)		

**b**							
	**Full sample**	**Differential concern groups**				**Chi-square**	**p**
		**Medium concern, full adaptation**	**Medium concern, some adaptation**	**High concern, some adaptation**	**High concern, no adaptation**		
*Sample size* (*N*)	765	153	262	257	93		
*Gender* Female *N* (%)	408 (53%)	74 (48%)	144 (55%)	132 (51%)	58 (62%)	5.60	0.14
*Age* 10 + N (%)	419 (55%)	86 (56%)	150 (57%)	133 (52%)	50 (54%)	1.90	0.59
*COVID-19 vaccination* *N* (%)	160 (21%)	33 (22%)	55 (21%)	58 (23%)	14 (15%)	1.90	0.59
*Diagnosis* *N* (%)							
Gastroenterology	81 (11%)	11 (7%)	20 (8%)	40 (16%)	10 (11%)	11.00	0.01
Nephrology	88 (12%)	16 (10%)	38 (15%)	26 (10%)	8 (9%)	3.70	0.29
Solid organ transplant	27 (4%)	3 (2%)	6 (2%)	12 (5%)	6 (6%)	5.60*	0.14
Diabetes	23 (3%)	3 (2%)	10 (4%)	9 (4%)	1 (1%)	2.57*	0.55
Immune Deficient	81 (11%)	11 (7%)	36 (14%)	24 (9%)	10 (11%)	5.04	0.17
Oncology	54 (7%)	15 (10%)	11 (4%)	23 (9%)	5 (5%)	6.82	0.08
Respiratory	45 (6%)	10 (7%)	13 (5%)	17 (7%)	5 (5%)	0.81	0.84
Juvenile Idiopathic Arthritis	282 (37%)	62 (41%)	97 (37%)	86 (33%)	37 (40%)	2.50	0.48
Other immune	134 (18%)	28 (18%)	58 (22%)	37 (14%)	11 (12%)	7.75	0.05
Other	188 (25%)	34 (22%)	59 (23%)	67 (26%)	28 (30%)	2.90	0.41
*Medications* *N* (%)							
Other drugs^c^	353 (46%)	70 (46%)	116 (44%)	123 (48%)	44 (47%)	0.73	0.87
Chemotherapy	4 (1%)	1 (1%)	3 (1%)	0 (0%)	0 (0%)	3.84*	0.32
Antibiotics	121 (16%)	20 (13%)	47 (18%)	39 (15%)	15 (16%)	1.84	0.61
Other immune suppressants	166 (22%)	27 (18%)	57 (22%)	65 (25%)	17 (18%)	4.07	0.25
Corticosteroids	152 (20%)	31 (20%)	49 (19%)	61 (24%)	11 (12%)	6.43	0.09
Biologics	236 (31%)	56 (37%)	81 (31%)	74 (29%)	25 (27%)	3.57	0.31
Methotrexate	216 (28%)	51 (33%)	67 (26%)	68 (26%)	30 (32%)	4.02	0.26
*Location medical care N* (%)							
South East	191 (25%)	40 (26%)	73 (28%)	54 (21%)	24 (26%)	3.47	0.33
Southwest	25 (3%)	2 (1%)	6 (2%)	13 (5%)	4 (4%)	5.57	0.13
East	48 (6%)	4 (3%)	11 (4%)	21 (8%)	12 (13%)	13.93	0.004
East Midlands	47 (6%)	13 (8%)	14 (5%)	12 (5%)	8 (9%)	3.70	0.30
West Midlands	142 (19%)	25 (16%)	49 (19%)	57 (22%)	11 (12%)	5.52	0.14
Yorkshire and Humber	31 (4%)	11 (7%)	10 (4%)	10 (4%)	0 (0%)	7.86	0.03
Northwest	87 (11%)	18 (12%)	36 (14%)	27 (11%)	6 (6%)	3.91	0.27
Northeast	156 (20%)	31 (20%)	55 (21%)	51 (20%)	19 (20%)	0.11	0.99
Wales	35 (5%)	8 (5%)	7 (3%)	12 (5%)	8 (9%)	5.78	0.12

*Represents the *P* value of a Fisher exact test; **represents *P* value for Kruskal–Wallis statistic.

^c^Other drugs include inhalers, eye drops, NSAIDs, folic acid, hypertonic saline, Omeprazole, insuline, hydrochloroquine, colchicine, antihypertensives, IViG, leflunomide, sulfasalazine, and mercaptopurine.

### Trajectories of concern

Our first step in the trajectory analysis was to determine the number of different trajectories that best represent parental concern over the first 18 months of the COVID-19 pandemic. Table 2 shows that the BIC and AIC values improve with increasing number of trajectories. A Bayes factor comparing the BIC of the simpler model with the more complex model (2(ΔBIC)) was calculated to determine the best fitting model. A 10-fold difference in Bayes factor was considered strong evidence that the more complex model best represented the data^19^. Convergence of the 5-trajectory model failed as the PROC TRAJ procedure found it difficult to estimate a 5-trajectory mathematical model among the mixture of parental concern trajectories provided using the likelihood estimation methodology employed by the procedure. It was concluded that the 4-trajectory model best fitted the data. The average posterior probability for the 4 different concern trajectories was well above the recommended minimum average posterior probability of 0.7 (ranged between 0.9969 and 0.9891), indicating that each pattern includes individuals with quite similar patterns of change in parental concern. In addition, the four distinct trajectories represented change in concern over time for parental group whose size was well over the 5% sample size threshold. The four trajectories represent (1) parents (20.1%) with medium levels of concern at the start of the COVID-19 pandemic but full adaptation over the next 18 months (2) parents (34.2%) with medium levels of concern at the start of the COVID-19 pandemic and some adaptation over the next 18 months (3) parents (34%) with high levels of concern at the start of the COVID-19 pandemic and some adaptation over the next 18 months (4) parents (11.7%) who experienced high levels of concern throughout the 18 months of the COVID-19 pandemic. Household and child characteristics as well as univariate comparisons between these four trajectory groups are presented in Table 1a, b.

**Table 2:** Determination of best fitting model for parental concern during first 18 months of COVID-19 pandemic

	Model	BIC	2(ΔBIC)	AIC	Sample distribution	Average Posterior Probability
First Phase: quadratic polynomial	1 trajectory	−92009.31		−92000.03	100%	
	2 trajectory	−76840.30	30338.02	−76821.74		
	Group 1				47.3%	0.9956
	Group 2				52.7%	0.9942
	3 trajectory	−69036.96	15606.68	−69009.12		
	Group 1				29.2%	0.9975
	Group 2				51.4%	0.9972
	Group 3				19.4%	0.9963
	4 trajectory	−63998.14	10077.64	−63961.02		
	Group 1				20.5%	0.9969
	Group 2				34.1%	0.9940
	Group 3				33.8%	0.9924
	Group 4				11.6%	0.9891
	5 trajectory	−62258.22	3479.84	−62211.82		
	Group 1				11.1%	0.9999
	Group 2				19.6%	0.9999
	Group 3				30.1%	0.9964
	Group 4				29.9%	0.9951
	Group 5				9.2%	0.9905
Second phase: 2 mixed polynomial	4 trajectory	−63907.49		−63865.73		
	Group 1—cubic				20.1%	0.9958
	Group 2—cubic				34.2%	0.9955
	Group 3—quadratic				34.0%	0.9953
	Group 4—quadratic				11.7%	0.9927

**Fig. 1:**
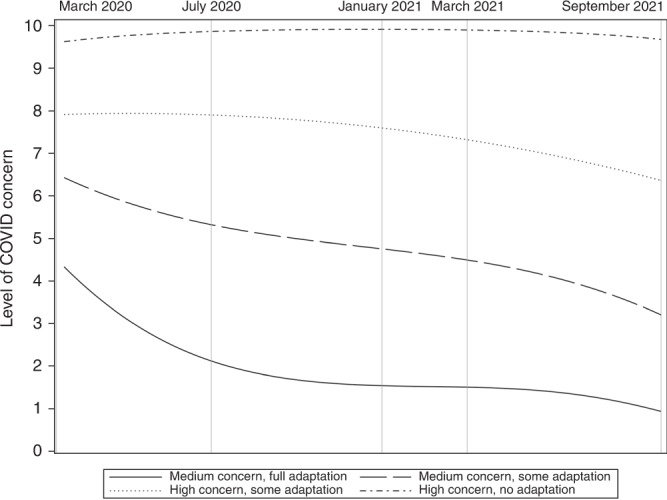
Parental concern during the first 18 months of the COVID-19 pandemic.

The second step in the trajectory analysis was to determine the polynomial growth terms that best represented the true shape of the trajectories. For each of the different trajectories we compared linear, quadratic, and cubic shapes. Based on the significance level of the estimated trajectory parameters, the medium parental concern groups were best represented (Fig. 1) by a cubic trajectory shape represented by decreasing parental concern after the initial concern response at the beginning of the pandemic, followed by increased concern during the second lockdown and decrease of concern during the roadmap out of lockdown. The high parental concern groups were best represented by a quadratic shape of slightly increasing parental concern after initial response at the beginning of the pandemic followed by a decrease of concern during the roadmap out of lockdown.

In the third step of the trajectory analysis, we examined the association of time-stable covariates and parental concern trajectory membership by expanding the PROC TRAJ syntax with the ‘RISK’ statement. Significant associations are presented in Table 3. In these analyses the ‘medium concern—full adaptation’ group was chosen as the reference group. No differences were found between the four parental concern trajectory groups with regard to their immunosuppressed child becoming infected with SARS-CoV-2, the number of self-isolations, or missing out on school, sport/leisure activities.

**Table 3:** Factors that significantly influence the probability of belonging to a parental concern trajectory

Household/personal characteristic	Medium concern, full adaptation	Medium concern, some adaptation	High concern, some adaptation	High concern, no adaptation
(semi) Rural	Reference category	*0.58* (0.29)*	*0.30* (0.28)	*0.31* (0.38)
Single parent		*−0.77* (0.39)*	*−1.13* (0.48)***	*−0.07* (0.46)
Pt/ft employment		*−0.07* (0.41)	*−0.69* (0.39)	*−1.58* (0.45)***
Living in North of England		*0.57* (0.35)	*0.29* (0.37)	*1.23* (0.42)***
Treatment—Northeast England		*0.02* (0.41)	*0.42* (0.39)	*1.55* (0.41)***
Treatment—Southwest England		*1.48* (0.76)	*1.61* (0.76)*	*0.82* (0.82)
Treatment—Southeast England		*−0.06* (0.26)	*−0.65* (0.28)*	*−1.00* (0.42)*
Child—nephrotic disease		*0.54* (0.40)	*0.35* (0.41)	*1.12* (0.44)*
Child—organ transplant		*−0.28* (0.43)	*0.32* (0.40)	*1.28* (0.43)***
Child—other primary immune deficiencies^a^		*−0.22* (0.43)	*0.75* (0.38)*	*0.54* (0.46)
Child—respiratory disease		*0.71* (0.48)	*1.05* (0.46)*	*1.28* (0.52)*
Child—JIA		*−0.16* (0.21)	*−0.30* (0.21)	*−1.18* (0.32)***
Child—other immunosuppression^b^		*−0.08* (0.25)	*0.55* (0.24)*	*1.01* (0.29)***
Child—other immunosuppressants^c^		*0.13* (0.26)	*0.26* (0.26)	*0.90* (0.30)***
Child—other meds^d^		*0.25* (0.21)	*0.50* (0.21)*	*1.17* (0.28)***

The cells in this table represent *parameter estimate for the respective trajectory group* (Standard error for the parameter estimate) *p < 0.05; **P < 0.01; ***p < 0.001.

^a^diagnoses such as antibody or complement deficiency, cellular immunodeficiency, or hyposplenia.

^b^Diagnoses such as Trisomy-21, severe eczema, cardiac disorder with abnormal immune function, neurological disorder, and haematological disorder

^c^Other immune suppressants like doxycycline, azathioprine, and tacrolimus.

^d^Other medications such as inhalers, eye drops, NSAIDs, folic acid, hypertonic saline, Omeprazole, insuline, hydrochloroquine, colchicine, antihypertensives, IViG, leflunomide, sulfasalazine, mercaptopurine.

Compared to the ‘medium concern—full adaptation’ group, parents in the ‘18 months high concern’ group were significantly less likely to be employed (*p* < 0.001) and more likely to live in the North of England (*p* < 0.01), more specifically their child was more likely to receive medical care in North-East England (*p* < 0.001), and significantly less likely to receive medical care in South-East England (*p* < 0.05). In addition, these parents were significantly more likely to care for children with nephrotic disease (*p* < 0.05), organ transplants (*p* < 0.01), respiratory diseases (*p* < 0.05), or children with diagnoses such as Trisomy-21, severe eczema, cardiac disorder with abnormal immune function, neurological disorder, haematological disorder (*p* < 0.001), and significantly less likely to care for children with Juvenile Idiopathic Arthritis (*p* < 0.001). These condition associations were mirrored by associations with the different drugs used in each condition (*p* < 0.05 to *P* < 0.001).

Compared to the ‘medium concern—full adaptation’ group, parents in the ‘high concern—some adaptation’ group were significantly less like to be single parents (*p* < 0.001), and more likely to care for children with other primary immunodeficiencies (*p* < 0.05), respiratory diseases (*p* < 0.05), children with diagnoses including Trisomy-21, eczema, cardiac disorder, neurological disorders, haematology disorders (*p* < 0.05) or children who are prescribed drugs like inhalers, eye drops, NSAIDs, folic acid, hypertonic saline, Omeprazole, insuline, hydrochloroquine, colchicine, antihypertensives, IViG, leflunomide, sulfasalazine, mercaptopurine (*p* < 0.05).

Compared to the ‘medium concern—full adaptation’ group, parents in the ‘medium concern—some adaptation’ group were significantly less likely to be single parents (*p* < 0.05) and significantly more likely to live in rural or semi-rural areas of the UK (*p* < 0.05).

### Content of concern

Each week the parents were asked to describe their current concerns. The parental concerns for the first 18 months of the pandemic could be grouped into four overarching themes: impact on everyday life child; impact on household; SARS-CoV-2 risk; and long-term health condition. Under these overarching themes we identified several sub-themes (Table 4).

**Table 4:**
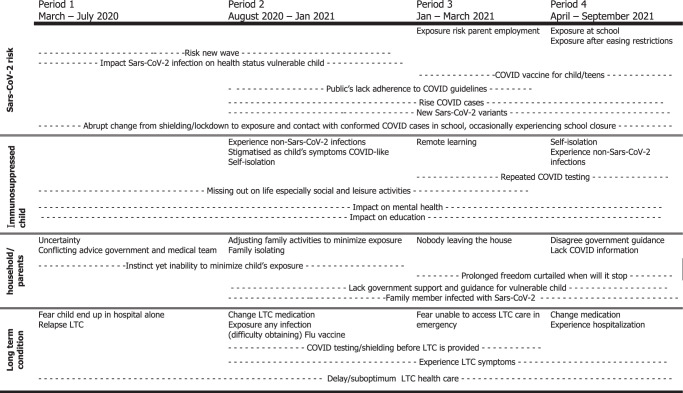
Most prevalent parental concerns during the COVID-19 pandemic.

During the study, parents expressed both general and COVID-19-specific concerns about their child’s health. They worried about the impact of (non-SARS-CoV-2) infections and childhood illnesses on the health status of their immunosuppressed child. Specific to the COVID-19 pandemic, parents of immunosuppressed children were worried about the impact of delay in diagnostic procedures, referral, and health care provision on their child’s health status, experiencing first-hand the effects of an overstretched NHS. Children needed to isolate for upcoming medical procedures and parents reported concerns that children were suffering psychologically from parental separation during these hospitalisations as UK hospital rules in general allowed only one parent visitor. Some children experienced flare-ups/exacerbation of the symptoms of their long-term condition, which parents sometimes contributed to COVID-related anxiety and stress or lack of exercise.

Throughout the pandemic, parents’ perceived risk of SARS-CoV-2 infection was in line with their perceptions of various SARS-CoV-2 strains, availability and perceived effectiveness of COVID-19 vaccines, prior SARS-CoV-2 infection, perceived effectiveness of preventative COVID-19 guidelines, and public adherence to COVID-19 guidelines. Parents were worried about the impact of a SARS-CoV-2 infection on the health status of their immunosuppressed child, specifically would COVID-19 cause serious illness or even death? As the pandemic progressed, the fear of death subsided, but a fear of long-term COVID-19 implications emerged.

Some parents mentioned exercising continual vigilance to minimise potential SARS-CoV-2 exposure both for the immunosuppressed child and any other vulnerable individuals living in the household. In general, parents believed that the life of their immunosuppressed child had been put on hold during the pandemic. Clinically vulnerable children and their families have experienced shielding and/or repeated periods of isolation. Parental concerns suggested these events impacted social development, education, and mental health. Families had to persist through uncertain times expressing concerns about the lack of information and inconsistent advice or guidelines. Some parents reported that they were more protective of their immunosuppressed child and have been weighing up the risk of SARS-CoV-2 exposure versus the benefits of social interaction, forsaking the social life of various household members if deemed necessary. As the COVID-19 pandemic persisted, some parents wondered what life would be like for a vulnerable child in the age of the COVID-19 pandemic? Families also reported having missed out on activities, holidays and social events, although this may be true of all children not just those immunosuppressed.

## Discussion

This is the first large-scale prospective cohort study to explore the concerns of parents caring for a vulnerable child during the COVID-19 pandemic. We identified four different patterns while exploring the level of concern experienced by parents caring for an immunosuppressed child during the first 18 months of the COVID-19 pandemic. As O’Connor et al.^20^ identified, we found that parental concern in the UK was high in the beginning of the COVID-19 pandemic. For some the high concern remained, but for most baseline concern gradually decreased as parents adapted to the changing circumstances. When new lockdowns were announced in November 2020 and January 2021 the concern level slightly increased again. Since the UK has entered the roadmap out of lockdown, the concern level has slowly decreased for all parents of immunosuppressed children. Although some studies announce that the distress level experienced by the UK population after the first lockdown were similar to pre-pandemic stress levels^21^ our data suggest this was not the case for parents caring for an immunosuppressed child. We note that the parents of immunosuppressed children were confused by the contradictory information received from government, researchers, doctors, and the media regarding the vulnerability of their child to SARS-CoV-2. Especially during the second lockdown, some parents were scared to send their child into school where there was a lack of social distancing. When vaccinations were rolled out to clinically vulnerable over 16s in July 2021, parental levels of concern reduced.

A number of different individual/household characteristics were related to the four differential parental concern trajectories. Northern England has been impacted more by the COVID-19 pandemic, with increased mortality rates and more healthcare resources being employed for the COVID-response compared to the Southern areas. In addition, people living in North England spent more time in restrictive lockdown than people living elsewhere in the UK^22^. These factors are likely to have impacted the level of parental concern. The findings reveal that non-working parents were more likely to have had high levels of concern during the pandemic. While working from home and home-schooling created competing time-demands on parents, and especially single parents during the pandemic^23^, the economic shock and financial demands of home-schooling might have impacted the overall concerns of non-working parents due to their lack of financial safeguards^24^.

Our findings also provide valuable insight into the sources of concern for parents caring for an immunosuppressed child during the COVID-19 pandemic. Consistent with the reports from other UK parents^25,26^, parents caring for an immunosuppressed child voiced the social and mental health impact of the pandemic on the child’s life as well as the continuous vigilance to protect the vulnerable child from potential SARS-CoV-2 exposure. Repeated life adjustments were needed to adapt to the ever-changing new COVID-19 pandemic normality.

### Clinical implications

Parental concern is a window into psychological wellbeing of families living with vulnerable children. The high level of concern reported by all parents at the start of the pandemic points to a need for targeted psychological support for this population in future pandemics. Results suggest that an uncertainty regarding government advice influenced the level of concern, therefore medical teams supporting these families need to react with targeted, clear and timely advice targeted to the individual child, in order to minimise parental levels of concern. To reduce concern levels, parents would benefit from consistent messaging from national and local parts of the UK health system.

Parents may also benefit from facilitated parent support groups, where they could receive access to reliable, consistent medical knowledge and have space to connect with other families undergoing similar experiences. Interventions with an Acceptance and Commitment Therapy (ACT) approach may also be useful for this population as it has been reported to be effective in reducing parental distress and increasing psychological flexibility in parents of children with a chronic health condition^27^. By focusing on increasing psychological flexibility and engagement in meaningful and valued activities, ACT may be useful for helping parents balance the understandable worries regarding the impact of the COVID-19 pandemic on their child. This is true especially in the context of distress or concern that cannot be avoided or minimised, whilst also considering how their vulnerable child can live a valued and meaningful life in spite of these challenges.

Long-term follow-up of the 11.7% of parents with a continuous very high level of concern is needed to fully understand whether this represents pre-pandemic levels of concern or are a direct result of the pandemic. The finding that almost half of the parents expressed high to very high levels of concern throughout the pandemic indicates a need for routine psychological screening of families with immunosuppressed children, to identify areas of emotional need throughout the child’s medical journey and to better inform future psychological support for the most affected.

The concerns raised by parents regarding the social impact of the lockdowns and restrictions may mirror existing concerns about their child already being vulnerable to missing out on social activities, due to hospital appointments, medical procedures and missing school due to illness. This could indicate that this population might benefit from targeted peer group support provision, especially at the time of national epidemics or pandemics, which have the potential to further isolate and alienate this group.

The results suggesting geographical factors associated with specific patterns of concern indicate that local NHS integrated health systems could tailor their intervention to the specific needs of their population, and in order to do this, routine local psychological screening will be needed.

### Limitations

Despite the large sample size, the ImmunoCOVID-19 study did not use a random sampling frame to recruit the parents of immunosuppressed children in the UK, as participants were recruited via specialists at 46 NHS UK institutions. Specialists referred families to the study team who sent them an invitation to participate which could have introduced a selection bias.

This study focused on parental concern experienced during the first 18 months of the COVID-19 pandemic. No data was collected on parental concern for these participants under usual circumstances, before the pandemic. It is therefore an exploratory study, not able to be compared to normal or control data.

The Latent Class Growth modelling technique used in this study has allowed us to identify four different types of concern trajectories experienced by parents caring for an immunosuppressed child during the first 18 months of the COVID-19 pandemic as well as factors influencing group membership. It was however out of the scope of this exploratory project to study interaction effects (e.g. potential reciprocal relationship between parental concern and reporting of SARS-CoV-2 infection). The underlying assumption of the methodology used is that the missing data for this cohort is missing at random. Deviations from this assumption could potentially introduce bias^28^.

## Conclusion

This study provides initial insights regarding the concern response of parents caring for immunosuppressed children to the challenges posed by the COVID-19 pandemic. The concern trajectories of parents experiencing medium concern paralleled changes in pandemic guidance and rates of SARS-CoV-2 infection in the UK. However, a substantial number of parents showed high levels of concern with little sign of adaptation. Parents caring for a child diagnosed with nephrotic or respiratory disease, a child on the organ transplant waiting list, parents who were not employed, and parents living in the North of England were at greater risk of higher levels of concern. Parents caring for children with Juvenile Idiopathic Arthritis and receiving medical care in the South of England were at lower risk for high concern levels. Clinical awareness of the parents that are at greater risk for high concern levels will allow clinicians to implement appropriate interventions and guidance for parents caring for an immunosuppressed child. Future research should focus on the long-term effects on vulnerable child and their parents.

## Supplementary Information

Appendix A

## Data Availability

Due to the quick deployment of the ImmunoCOVID-19 study we have not actively asked for participants’ permission to safely deposit their data for re-share and re-use, thus while quantitative analysis code can be shared the data for this project cannot be deposited.
