# Supplementary material for: Parental concern for clinically vulnerable child during first 18 months of the COVID pandemic
Source: Pediatr Res. 2022 Nov 22;94(1):222–30. doi: 10.1038/s41390-022-02371-7 (PMC9684768; doi:10.1038/s41390-022-02371-7)
Supplement: Supplementary file 1 — Appendix A [file 41390_2022_2371_MOESM1_ESM.pdf]

Appendix A: List of ImmunoCOVID19 Study Group Co-authors

Dr	Hesham Abdalla	Consultant Paediatrician	Walsall Healthcare NHS Trust	Moat Road, Walsall WS2 9PA
Ms	Daniella Adams	Clinical Nurse Specialist Paediatric Rheumatology	Evelina London Children's Hospital, Guy's and St Thomas' NHS Foundation Trust	Westminster Bridge Road London, SE1 7EH
Dr	Nadeem A Afzal	Consultant Paediatric Gastroenterologist	Department of Paediatric Gastroenterology, Southampton Children's Hospital	Tremona Road, Southampton, SO16 6YD
Mr	Nicolas Aldridge	Lead Nurse R and D	Birmingham Women's and Children's Hospital and University Hospital Coventry and Warwickshire	2nd Floor, PRI suit, UHCW, Clifford Bridge Road, CV2 2DX
Dr	Paola Angelini	Consultant Paediatric Oncology	Royal Marsden Hospital	Downs Road, Sutton, SM2 7HE
Dr	Kate Armon	Paediatric Rheumatologist	Paediatric Department, Addenbrookes hospital, Cambridge	Box 267 Children's Services, Biomedical Campus, hills Road, Cambridge Cb2 0QQ
Dr	James Ashton	Clinical Research Fellow in Paediatric IBD	Human Genetics and Genomic Medicine, University of Southampton, Department of Paediatric Gastroenterology, Southampton Children's Hospital	Tremona Road, Southampton, SO16 6YD
Mrs	Helen Baker	Data Manager	Paediatric Oncology and Haematology, Cambridge University Hospitals NHS Foundation Trust	Hills Road, Cambridge, CB2 0QQ
Mrs	Nicola Balatoni	Research Nurse	Leeds Children Hospital	Leeds Teaching Hospital NHS Trust, Great George Street, LS1 3EX
Dr	Jed Bamber	Consultant Paediatrician	NHS Dumfries and Galloway	Dumfries & Galloway Royal Infirmary, Cargenbridge, Dumfries, DG2 8RX
Mrs	Alison Barratt	Paediatric Clinical Research Nurse	Royal United Hospitals Bath NHS Foundation Trust	Combe Park, Bath, BA1 3NG
Mrs	Charlene Bass-Woodcock	Paediatric Rheumatology Clinical Nurse Specialist	Bradford Teaching Hospitals NHS Foundation Trust	Duckworth Lane, Bradford, West Yorkshire, BD9 6RJ
Dr	Jessica Bate	Consultant Paediatric Oncology	Department of Paediatric Oncology, University Hospital Southampton NHS Foundation Trust	Tremona Road, Southampton, SO16 6YD
Dr	Akshay Batra	Consultant Paediatric Gastroenterologist	Department of Paediatric Gastroenterology, Southampton Children's Hospital	Tremona Road, Southampton, SO16 6YD
Prof	R Mark Beattie	Consultant Paediatric Gastroenterologist	Department of Paediatric Gastroenterology, Southampton Children's Hospital	Tremona Road, Southampton, SO16 6YD
Dr	Rumena Begum	Research Coordinator	Great Ormond Street Hospital for Children NHS Foundation Trust	Great Ormond Street, London WC1N 3JH
Miss	Claire Bell	Paediatric Research Nurse	University Hospital Crosshouse	Kilmarnock, KA2 0BE
Ms	Kathryn Bell	Research Nurse	Great North Children's Hospital	Royal Victoria Infirmary, Newcastle upon Tyne, NE1 4LP
Dr	Georgina Bird-Lieberman	Paediatric Neurology Consultant	University Hospital Southampton NHS Foundation Trust	Tremona Road, Southampton, SO16 6YD
Ms	Helen Blackburn	Research and Innovation Manager	Royal Manchester Children's Hospital, Manchester University Hospitals NHS Trust,	Royal Manchester Children's Hospital, Manchester University Hospitals NHS Trust, Oxford Rd Manchester M13 9WL UK
Dr	Mary Brennan	Consultant Paediatric Rheumatologist	Royal Hospital for Sick Children, Edinburgh	9 Sciennes Road, Edinburgh, EH1 9LF
Ms	Francesca Brewer	Research sister : Paediatrics and Neonates	Birmingham Women's and Children's Hospital and University Hospital Coventry and Warwickshire	R&D Clinical Trials Office, 3rd Floor, West wing, UHCW, Clifford Bridge, CV2 2DX
Mrs	Marlene Brito	Clinical Trials Assistant	Department of Paediatric Oncology, University Hospital Southampton NHS Foundation Trust and Faculty of Medicine and Institute for Life Sciences, University of Southampton	Tremona Road, Southampton, SO16 6YD
Dr	Richard Brough	Consultant urologist and urological cancer lead	The Shrewsbury and Telford Hospital NHS Trust	Mytton Oak Road, Shrewsbury, Shropshire, SY3 8XQ

Dr	Susan Byrne	Paediatric Neurology Consultant	Evelina London Children' Hospital	Westminster Bridge Road, London, SE1 7EH
Dr	Katrina Cathie	Consultant General Paediatrics and Paediatric Research	University Hospital Southampton NHS Foundation Trust	Tremona Road, Southampton, SO16 6YD
Dr	John Chapman	Consultant Paediatrician	James Paget University Hospital	Lowestoft Rd, Gorleston, NR31 6LA
Dr	Harry Chappell	Academic Foundation Doctor	University Hospital Southampton NHS Foundation Trust	Tremona Road, Southampton, SO16 6YD
Dr	Anne-Marie Child	Consultant Paediatric Neurologist	Leeds Children Hospital	Leeds Teaching Hospital NHS Trust, Great George Street, LS1 3EX
Dr	Coziana Ciurtin	Consultant Adolescent Rheumatology	Centre for Adolescent Rheumatology Versus Arthritis, University College London	3rd Floor Central, 250 Euston Road, London, NW1 2PG
Dr	Sheila Clark	Consultant dermatology	Leeds Children Hospital	Leeds Teaching Hospital NHS Trust, Great George Street, LS1 3EX
Dr	Tracy Coelho	Consultant Paediatric Gastroenterologist	Department of Paediatric Gastroenterology, Southampton Children's Hospital	Tremona Road, Southampton, SO16 6YD
Dr	William Coles	Consultant Paediatric Rheumatologist	Birmingham Women's and Children's Hospital and University Hospital Coventry and Warwickshire	Paediatric Rheumatology, Paediatric Department, UHCW, Clifford Bridge, CV2 2DX
Mrs	Heather Collier	Research Nurse	East Lancs NHS Trust	Haslingden Road, Blackburn, BB2 3HH
Dr	Gary Connett	Paediatric Respiratory Consultant	University Hospital Southampton NHS Foundation Trust	Tremona Road, Southampton, SO16 6YD
Dr	Philip Connor	Consultant Paediatric Haematology	Noah's Ark Children's Hospital For Wales	Rm 1F31 Cardigan House, UHW, Cardiff, CF14 4XW
Dr	Michael Cosgrove	Consultant Paediatric Gastroenterologist	Morriston Hospital, Swansea Bay University Health Board	Swansea, SA6 6NL
Ms	Gillian Coyle	Clinical Nurse Specialist	Royal Hospital for Sick Children, Edinburgh	9 Sciennes Road, Edinburgh, EH1 9LF
Dr	Brian Davidson	Consultant Rheumatologist	University Hospital Southampton NHS Foundation Trust	Tremona Road, Southampton, SO16 6YD
Mrs	Matthew Davies	Data Manager	Department of Neonatal Medicine, University Hospital Southampton NHS Foundation Trust	
Mrs	Sharon Davies-Dear	Clinical Trials Project Manager	R&D, University Hospital Southampton NHS Foundation Trust	Tremona Road, Southampton, SO16 6YD
Dr	Christian De Goede	Consultant Paediatric Neurologist	Department of Child Health, Lancashire Teaching Hospitals NHS Trusts	Royal Preston Hospital, Sharoe Green Lane, Preston PR2 9HT
Dr	Hans de Graaf	Consultant Paediatric Rheumatology	NIHR Southampton Clinical Research Facility and Paediatric Medicine, University Hospital Southampton NHS Foundation Trust	Tremona Road, Southampton, SO16 6YD
Dr	Samundeeswari Deepak	Consultant Paediatric and Adolescent rheumatology	Nottingham Children's Hospital, Queen's Medical Centre, Nottingham University Hospitals NHS Trust	Derby Road, Nottingham, NG72UH
Mr	Fatjon Dekaj	CLUSTER Research Coordinator	Infection, Immunity and Inflammation Research & Teaching Department, University College London Great Ormond Street Institute of Child Health	30 Guilford Street, London, WC1N 1EH
Miss	Sarah Diment	Paediatric Research Nurse	Salisbury District Hospital	Salisbury, Wiltshire, SP2 8BJ
Dr	Corine Driessens	Research Fellow	University of Southampton, Faculty of Medicine, Clinical and Experimental Sciences	Tremona Road, Southampton, SO16 6YD
Dr	Simon B Drysdale	Consultant in Paediatric Infectious Diseases and Immunology	Paediatric Infectious Diseases Research Group, St George's, University of London and St George's University Hospitals NHS Foundation Trust	Cranmer Terrace, London, SW17 0RE
Dr	Martin Elliott	Consultant paediatric Oncology	Leeds Children Hospital	Leeds Teaching Hospital NHS Trust, Great George Street, LS1 3EX
Prof	Marieke Emonts	Consultant Paediatric Infectious Diseases &	Newcastle Hospitals NHS Foundation Trust; Translational and Clinical Research Institute, Newcastle University, and NIHR Newcastle	Royal Victoria Hospital, Queen Victoria Road, Newcastle upon Tyne. NE1 4LP

		Immunology, Honorary Professor Paediatric Infectious Diseases	Biomedical Research Centre based at Newcastle upon Tyne Hospitals NHS Trust and Newcastle University	
Dr	Mich Erlewyn-Lajeunesse	Consultant Paediatric Allergy and Immunology	University Hospital Southampton NHS Foundation Trust	Tremona Road, Southampton, SO16 6YD
Dr	Hazel Evans	Paediatric Respiratory Consultant	University Hospital Southampton NHS Foundation Trust	Tremona Road, Southampton, SO16 6YD
Mrs	Tracey Farnon	Paediatric Consultant	Salisbury District Hospital	Salisbury, Wiltshire, SP2 8BJ
Prof	Saul Faust	Professor of Paediatric Immunology and Infectious Diseases	NIHR Southampton Clinical Research Facility and NIHR Southampton Biomedical Research Centre, University Hospital Southampton NHS Foundation Trust and Faculty of Medicine and Institute for Life Sciences, University of Southampton	Tremona Road, Southampton, SO16 6YD
Dr	Susanna Felsenstein	Consultant Paediatric Infectious Diseases	Alder Hey Children’s Hospital	Liverpool, East Prescot Rd L14 5AB
Ms	Lynsey Felton	Paediatric Research Nurse	James Paget University Hospital	Lowestoft Rd, Gorleston, NR31 6LA
Dr	Matthew Fenton	Consultant Paediatric Cardiologist	Great Ormond Street Hospital for Children NHS Foundation Trust	Great Ormond Street, London WC1N 3JH
Dr	Katy Fidler	Clinical Senior Lecturer in Paediatric Infectious Diseases and Immunology	Academic Dept of Paediatrics, Brighton and Sussex Medical School, University of Sussex	N - S Rd, Falmer, Brighton BN1 9PX
Mrs	Debbie Fisher	Trial Coordinator	Paediatric Oncology and Haematology, Cambridge University Hospitals NHS Foundation Trust	Hills Road, Cambridge, CB2 0QQ
Dr	Peter W Fowlie	Consultant Paediatrician	Ninewells Hospital and Medical School	Dundee, DD1 9SY
Mrs	Kirsty Galvin	Lead Nurse	Children's Community Nursing Team, Kent community Health Foundation Trust	Trinity House, 110-12- Upper Pemberton, Kennington, Ashford, Kent, TN25 4AZ
Ms	Emma Gardiner	Research Nurse	University Hospital Lewisham	Paediatric Offices, Nockold House, University Hospital Lewisham, Lewisham High street, London, SE13 6LH
Dr	Edward Gaynor	Paediatric Gastroenterology Consultant	Great Ormond Street Hospital for Children NHS Foundation Trust	Great Ormond Street, London WC1N 3JH
Dr	Diane Gbesemete	Clinical Research Fellow	NIHR Southampton Clinical Research Facility and NIHR Southampton Biomedical Research Centre, University Hospital Southampton NHS Foundation Trust and Faculty of Medicine and Institute for Life Sciences, University of Southampton	Tremona Road, Southampton, SO16 6YD
Dr	Rodney Gilbert	Consultant Paediatric Nephrologist	University Hospital Southampton NHS Foundation Trust	Tremona Road, Southampton, SO16 6YD
Dr	Francis J Gilchrist	Paediatric Respiratory Consultant	University Hospitals of North Midlands NHS Trust	Staffordshire Children’s Hospital at Royal Stoke, Newcastle Road, Stoke on Trent, ST4 6QG
Mrs	Clare Gilmour	Immunology Clinical Nurse Specialist	St George's University Hospitals NHS Foundation Trust	Cranmer Terrace, London, SW17 0RE
Ms	Claire Glemas	Research Nurse	Paediatric Gastroenterology, Cambridge University Hospitals NHS Foundation Trust	Hills Road, Cambridge, CB2 0QQ
Dr	Emma Grainger-Allen	Consultant Paediatric Allergy and Immunology	University Hospital Southampton NHS Foundation Trust	Tremona Road, Southampton, SO16 6YD
Dr	Juliet Gray	Associate Professor and Consultant in Paediatric Oncology	Department of Paediatric Oncology, University Hospital Southampton NHS Foundation Trust and Faculty of Medicine and Institute for Life Sciences, University of Southampton	Tremona Road, Southampton, SO16 6YD
Ms	Donna Griffiths	Paediatric Research Nurse	James Paget University Hospital	Lowestoft Rd, Gorleston, NR31 6LA
Mrs	Rowena Guermech	Research Nurse	Paediatric Oncology and Haematology, Cambridge University Hospitals NHS Foundation Trust	Hills Road, Cambridge, CB2 0QQ

Mr	Neil Hall	Research Nurse	Leeds Children Hospital	Leeds Teaching Hospital NHS Trust, Great George Street, LS1 3EX
Miss	Nicola Hamilton	Paediatric Rheumatology Link Nurse	NHS Forth Valley	Forth Valley Royal Hospital, Stirling Road, Larbert FK5 4WR
Mrs	Joanna Hancock	Clinical Nurse Specialist	University Hospital Southampton NHS Foundation Trust	Tremona Road, Southampton, SO16 6YD
Dr	Mushfequr R Haq	Consultant Paediatric Nephrologist	University Hospital Southampton NHS Foundation Trust	Tremona Road, Southampton SO16 6YD
Mrs	Amanda Harris	PCD & Children's Respiratory Clinical Nurse Specialist	NIHR Southampton Biomedical Research Centre, University Hospital Southampton NHS Foundation Trust	
Dr	Kathryn Harrison	Consultant Paediatric & Adolescent Rheumatology	Rheumatology Department, Birmingham Children's Hospital, Birmingham Women's and Children's NHS Foundation Trust	Steelhouse Lane, Birmingham B4 6NH
Ms	Rebecca Harrop	Data Manager	Great North Children's Hospital	Royal Victoria Infirmary, Newcastle upon Tyne, NE1 4LP
Miss	Sarah Hartfree	Paediatric Rheumatology Clinical Nurse Specialist	University Hospital Southampton NHS Foundation Trust	Tremona Road, Southampton SO16 6YD
Miss	Tyler Harvey-Cowlishaw	Laboratory Technician	School of Life Sciences, University of Nottingham	Nottingham, NG7 2RD
Mrs	Laura Hennelly	Adolescent Research Nurse	Centre for Adolescent Rheumatology Versus Arthritis, University College London	3rd Floor Central, 250 Euston Road, London, NW1 2PG
Dr	Avni Hindocha	Paediatric trainee	Royal Manchester Children's Hospital, Manchester University Hospitals NHS Trust,	Royal Manchester Children's Hospital, Manchester University Hospitals NHS Trust, Oxford Rd Manchester M13 9WL UK
Dr	John Ho	Consultant Paediatrician	Whipps Cross University Hospital, London	Whipps Cross Road, Leytonstone, London, E11 1NR
Mrs	Ruth Howman	Clinical Research Sister	Birmingham Children's Hospital, Birmingham Women's and Children's NHS Foundation Trust	Steelhouse Lane, Birmingham B4 6NH
Ms	Camilla Huber	Research & Development	Birmingham Women's Hospital, Birmingham Women's and Children's NHS Foundation Trust	Mindelsohn Way, Birmingham B15 2TG
Dr	Marjorie Illingworth	Paediatric Neurology Consultant	University Hospital Southampton NHS Foundation Trust	Tremona Road, Southampton, SO16 6YD
Dr	Winnie Ip	Honorary Senior Lecturer & Consultant Paediatric Immunologist	Great Ormond Street Hospital for Children NHS Foundation Trust, Molecular and Cellular Immunology, University College London Great Ormond Street Institute of Child Health	Great Ormond Street, London WC1N 3JH, 30 Guilford Street, London, WC1N 1EH
Dr	Javed Iqbal	Consultant Paediatrician	East Lancs NHS Trust	Haslingden Road, Blackburn, BB2 3HH
Prof	Will Irving	Professor and Honorary Consultant in Virology	University of Nottingham and Nottingham University Hospitals NHS Trust.	Department of Microbiology, University Hospital, Queen's Medical Centre, Nottingham, NG7 2UH
Miss	Hannah Jackson	PhD student	School of Life Sciences, University of Nottingham	Nottingham, NG7 2RD
Dr	Mark J Johnson	Consultant Neonatologist	Department of Neonatal Medicine, University Hospital Southampton NHS Foundation Trust, NIHR Biomedical Research Centre, University of Southampton	Tremona Road, Southampton, SO16 6YD
Dr	Christine E Jones	Consultant Paediatric Infectious Diseases	University Hospital Southampton NHS Foundation Trust	Tremona Road, Southampton, SO16 6YD
Mr	James Jones	Paediatric Research Nurse	The Shrewsbury and Telford Hospital NHS Trust	Mytton Oak Road, Shrewsbury, Shropshire, SY3 8XQ
Dr	Jochen Kammermeier	Consultant Paediatric Gastroenterologist	Evelina London Children's Hospital	Westminster Bridge Road London SE1 7EH
Dr	Dagmar Kastner-Cole	Consultant Paediatrician	Tayside Children's Hospital, Ninewells Hospital and Medical School	Dundee, DD1 9SY
Dr	Akhila Kavirayani	Consultant Paediatric Rheumatology	Oxford University Hospitals NHS Foundation Trust	Nuffield Orthopaedic Centre, Oxford, OX3 7HE

Prof	Deirdre Kelly	Consultant Paediatric Hepatologist	Liver Unit, Birmingham Women's & Children's NHS Foundation Trust, University of Birmingham	Steelhouse Lane, Birmingham B4 6NH, Edgbaston, Birmingham B15 2TT
Ms	Imogen Kelly	Clinical Nurse Specialist	Royal Hospital for Sick Children, Edinburgh	9 Sciennes Road, Edinburgh, EH1 9LF
Dr	Ciara Kennedy	Clinical Trials Coordinator	Great North Children's Hospital	Royal Victoria Infirmary, Newcastle upon Tyne, NE1 4LP
Dr	Larissa Kerecuk	Clinical Research Specialty Lead Paediatrics, NIHR CRN West Midlands, Consultant Paediatric Nephrologist	Birmingham Children's Hospital, Birmingham Women's and Children's NHS Foundation Trust	Steelhouse Lane, Birmingham B4 6NH
Mr	Charles Keys	Consultant Paediatric Surgery	University Hospital Southampton NHS Foundation Trust	Tremona Road, Southampton, SO16 6YD
Miss	Aline Kimonyo	Research Coordinator	University College London Great Ormond Street Institute of Child Health	UCL Great Ormond Street Institute of Child Health 30 Guilford Street London WC1N 1EH
Mrs	Sharon King	Research Nurse	Great North Children's Hospital	Royal Victoria Infirmary, Newcastle upon Tyne, NE1 4LP
Mrs	Vicky King	Clinical Trials Practitioner	Salisbury District Hospital	Salisbury, Wiltshire, SP2 8BJ
Dr	Fenella Kirkham	Paediatric Neurology Consultant	University Hospital Southampton NHS Foundation Trust	Tremona Road, Southampton, SO16 6YD
Dr	Alice Leahy	Consultant Paediatric Rheumatologist	University Hospital Southampton NHS Foundation Trust	Tremona Road, Southampton SO16 6YD
Ms	Gemma Lee	Clinical Nurse Specialist – Paediatric IBD	Evelina London Children's Hospital	Westminster Bridge Road London SE1 7EH
Dr	Julian Legg	Paediatric Respiratory Consultant	University Hospital Southampton NHS Foundation Trust	Tremona Road, Southampton, SO16 6YD
Dr	Valentina Leone	Consultant Paediatric Rheumatology	Leeds Children Hospital	Leeds Teaching Hospital NHS Trust, Great George Street, LS1 3EX
Dr	Derek Lim	Consultant Clinical Geneticist	Clinical Genetics Department, West Midlands Regional Genetics Service, Birmingham Women's and Children's Hospital NHS Foundation Trust	Mindelsohn Way, Birmingham B15 2TG
Mrs	Adine Logan	Research Nurse	East Lancs NHS Trust	Haslingden Road, Blackburn, BB2 3HH
Prof	Jane Lucas	Professor of Paediatric Respiratory Medicine	PCD Centre, School of Clinical and Experimental Sciences, Faculty of Medicine and Institute for Life Sciences, University of Southampton, NIHR Southampton Biomedical Research Centre, University Hospital Southampton NHS Foundation Trust;	Tremona Road, Southampton SO16 6YD
Dr	David Lynn	Consultant Paediatrician	NHS Forth Valley	Forth Valley Royal Hospital, Stirling Road, Larbert FK5 4WR
Mrs	Susan Macfarlane	Paediatric Research Nurse	Scottish Paediatric Research Network, Ninewells Hospital and Medical School	Dundee, DD1 9SY
Ms	Lydia Makusha	Neurology Specialist Nurse	Neurology Specialist Nurse, Neurology Department, Birmingham Children's Hospital, Birmingham Women's and Children's NHS Foundation Trust	Steelhouse Lane, Birmingham B4 6NH
Dr	Gulshan A Malik	Consultant Paediatrician	Royal Aberdeen Children hospital	Westburn road, Aberdeen Scotland, AB25 2ZG
Dr	Stephen D Marks	Reader and Consultant in Paediatric Nephrology	NIHR Great Ormond Street Hospital Biomedical Research Centre, University College London Great Ormond Street Institute of Child Health	30 Guilford Street, London WC1N 1EH
Dr	Verghese Mathew	Consultant Paediatrician	Hull University Teaching Hospitals NHS Trust	Anlaby Road, Hull, HU3 2JZ
Dr	Janet E McDonagh	Senior Lecturer in Paediatric and Adolescent Rheumatology	Versus Arthritis Centre for Epidemiology; Centre for MSK Research, University of Manchester; NIHR Biomedical Research Centre, Manchester University Hospital NHS Trust; Department of Paediatric	Centre for MSK Research, Stopford Building, University of Manchester Oxford Rd Manchester M13 9PT

			and Adolescent Rheumatology, Royal Manchester Children’s Hospital, Manchester University Hospitals NHS Trust	
Dr	Flora McErlane	Consultant Paediatric Rheumatologist and Associate Clinical Lecturer	Newcastle Hospitals NHS Foundation Trust and Institute of Health and Society, Newcastle University	Royal Victoria Hospital, Queen Victoria Road, Newcastle upon Tyne. NE1 4LP
Mrs	Ann McGovern	Senior Clinical Research Practitioner in Rheumatology	Royal Manchester Children’s Hospital, Manchester University Hospitals NHS Trust,	Royal Manchester Children’s Hospital, Manchester University Hospitals NHS Trust, Oxford Rd Manchester M13 9WL UK
Ms	Karen McIntyre	Paediatric Specialist Nurse	Ninewells Hospital and Medical School	Dundee, DD1 9SY
Dr	Ross McLean	Specialty Doctor in Paediatrics	NHS Lanarkshire	Kirklands, Fallside Road. Bothwell, G71 8BB
Dr	Paddy McMaster	Consultant in Paediatric Infectious Diseases	North Manchester General Hospital	Delauneys Road, Manchester, M8 5RB
Dr	Nabil Melhem	Consultant Paediatric Nephrologist	Department of Paediatric Nephrology, Evelina London Children’s Hospital, Guy’s & St. Thomas’ Foundation Hospitals NHS Trust	Westminster Bridge Road, London, SE1 7EH, UK
Ms	Dawn Metcalfe	Clinical Trials Associate	Great North Children's Hospital	Royal Victoria Infirmary, Newcastle upon Tyne, NE1 4LP
Ms	Danielle Miller	Children's Research Nurse	Oxford University Hospitals NHS Foundation Trust	John Radcliff hospital, WWLG1 room 10.15, Oxford, OX3 9DU
Ms	Lynne Mills	Information Analyst Advanced	NIHR Southampton Clinical Research Facility, University Hospital Southampton NHS Foundation Trust	Tremona Road, Southampton, SO16 6YD
Ms	Lisa Moyes	Paediatric Research Practitioner	Nottingham Children’s Hospital, Queen’s Medical Centre, Nottingham University Hospitals NHS Trust	Derby Road, Nottingham, NG72UH
Dr	Alasdair Munro	Clinical Research Fellow	NIHR Southampton Clinical Research Facility and NIHR Southampton Biomedical Research Centre, University Hospital Southampton NHS Foundation Trust and Faculty of Medicine and Institute for Life Sciences, University of Southampton	Tremona Road, Southampton, SO16 6YD
Miss	Olivia Murphy-Parry	Paediatric Rheumatology Clinical Nurse Specialist	University Hospital Southampton NHS Foundation Trust	Tremona Road, Southampton SO16 6YD
Dr	Mohamed Mutalib	Consultant paediatric gastroenterologist	Evelina London Children’ Hospital	Westminster Bridge Road, London< SE1 7EH
Dr	Arvind Nagra	Consultant Paediatric Nephrologist	University Hospital Southampton NHS Foundation Trust	Tremona Road, Southampton SO16 6YD
Dr	Sarveshni Naidoo	Consultant General Paediatrics	NHS Lanarkshire	Kirklands, Fallside Road. Bothwell, G71 8BB
Dr	Gary Nicolin	Consultant Paediatric Oncology	University Hospital Southampton NHS Foundation Trust	Tremona Road, Southampton, SO16 6YD
Dr	Maggie Nyirenda	Consultant Paediatrician	University Hospital Lewisham	Paediatric Offices, Nockold House, University Hospital Lewisham, Lewisham High street, London, SE13 6LH
Mrs	Grainne O’Connor	Senior Paediatric Research Nurse	North Manchester General Hospital	Delauneys Road, Manchester, M8 5RB
Dr	Sian O’Riordan	Consultant Paediatric Immunology and Infectious Diseases	Leeds Children Hospital	Leeds Teaching Hospital NHS Trust, Great George Street, LS1 3EX
Dr	Briget Oates	Paediatric Consultant	University Hospital Crosshouse	Kilmarnock, KA2 0BE
Dr	Daniel Owens	Clinical Research Fellow	NIHR Southampton Clinical Research Facility and NIHR Southampton Biomedical Research Centre, University Hospital Southampton NHS Foundation Trust and Faculty of Medicine and Institute for Life Sciences, University of Southampton	Tremona Road, Southampton, SO16 6YD
Ms	Krishna Panchal	Clinical Trials Support Officer	Research Department, Lancashire Teaching Hospitals NHS Trusts	Royal Preston Hospital, Sharoe Green Lane, Preston PR2 9HT

Ms	Sharon Parkes	Nephrology and Rare Disease Research Coordinator	Nephrology and Rare Disease Research Coordinator, R&D Department , Birmingham Women's and Children's NHS Foundation Trust	Steelhouse Lane, Birmingham B4 6NH
Mrs	Charlotte Passingham	Research Co-ordinator	Liver Unit, Birmingham Children's Hospital, Birmingham Women's and Children's NHS Foundation Trust	Steelhouse Lane, Birmingham B4 6NH
Mr	Ravin Patel	Medical Student	University of Southampton	University Rd, Southampton SO17 1BJ
Dr	Sanjay Patel	Consultant Paediatric Infectious Diseases	University Hospital Southampton NHS Foundation Trust	Tremona Road, Southampton, SO16 6YD
Dr	Margaret Peebles	Consultant Paediatrician	Ninewells Hospital and Medical School	Dundee, DD1 9SY
Dr	Salina Persand	Clinical Research Practitioner	Imperial College Healthcare NHS Trust and Imperial College London. Children's Clinical Research Facility	
Mrs	Sharon Peters	Paediatric Infectious Diseases Nurse Specialist	North Manchester General Hospital	Delauneys Road, Manchester, M8 5RB
Mrs	Charlotte Phillips	Team leader	Childrens community Nursing Team, Kent community Health Foundation Trust	Trinity House, 110-12- Upper Pemberton, Kennington, Ashford, Kent, TN25 4AZ
Mrs	Helen Pidgeon	Clinical Trials Assistant	Salisbury District Hospital	Salisbury, Wiltshire, SP2 8BJ
Mrs	Sue Power	Paediatric Research Nurse	Poole Hospital NHS Foundation Trust	Longfleet Rd, Poole, BH15 2JB
Dr	Evgenia Preka	Consultant Paediatric Nephrologist	University Hospital Southampton NHS Foundation Trust	Tremona Road, Southampton SO16 6YD
Ms	Vanessa Raimondo	Clinical Nurse Specialist	Royal Hospital for Sick Children, Edinburgh	9 Sciennes Road, Edinburgh, EH1 9LF
Dr	Jagadeesh Ramachandra	Consultant Paediatrician	Royal United Hospitals Bath NHS Foundation Trust	Combe Park, Bath, BA1 3NG
Dr	Ramya Ramanujachar	Consultant Paediatric Oncology	University Hospital Southampton NHS Foundation Trust	Tremona Road, Southampton, SO16 6YD
Miss	Pernille Rasmussen	Clinical Nurse Specialist Nephrology	Department of Paediatric Nephrology, Evelina London Children's Hospital, Guy's & St. Thomas' Foundation Hospitals NHS Trust	Westminster Bridge Road, London, SE1 7EH, UK
Dr	Trevor Richens	Consultant Paediatric Cardiologist	University Hospital Southampton NHS Foundation Trust	Tremona Road, Southampton, SO16 6YD
Dr	Valerie Rogers	Consultant Paediatric Rheumatologist	University Hospital Bristol NHS Foundation trust	Marlborough Street, Bristol, BS1 3NU
Dr	Erika Rojas-Jimenz	Paediatric Research Clinical Fellow	Poole Hospital NHS Foundation Trust	Longfleet Rd, Poole, BH15 2JB
Dr	Kevin Roman	Consultant Paediatric Cardiologist	University Hospital Southampton NHS Foundation Trust	Tremona Road, Southampton, SO16 6YD
Ms	Chloe Saad	Clinical Research Assistant	University Hospital Lewisham	Paediatric Offices, Nockold House, University Hospital Lewisham, Lewisham High street, London, SE13 6LH
Mr	Stephen Saich	CRF Clinical Research Project Manager	NIHR Southampton Clinical Research Facility, University Hospital Southampton NHS Foundation Trust	Tremona Road, Southampton, SO16 6YD
Ms	Rebecca Sarjeant	Research Nurse	Great North Children's Hospital	Royal Victoria Infirmary, Newcastle upon Tyne, NE1 4LP
Ms	Paula Saunderson	Clinical Trials Co-ordinator	Alder Hey Children's NHS Foundation Trust	Eaton Road, West Derby, Liverpool, L12 2AP
Miss	Francesca Schiavone	Clinical Nurse Specialist, Paediatric Gastroenterology	Morriston Hospital, Swansea Bay University Health Board	Swansea, SA6 6NL
Ms	Beatrice Selby	Clinical research coordinator	NIHR Southampton Clinical Research Facility, University Hospital Southampton NHS Foundation Trust	Tremona Road, Southampton SO16 6YD
Dr	Fiona Shackley	Consultant Paediatric Infectious Diseases	Sheffield Children's Hospital	Clarkson St, Broomhall, Sheffield S10 2TH
Ms	Jennifer Sharp	Research Nurse	Paediatric Research , Cambridge University Hospitals NHS Foundation Trust	Hills Road, Cambridge, CB2 0QQ

Dr	Meera Shaunak	Clinical Research Fellow	NIHR Southampton Clinical Research Facility, University Hospital Southampton NHS Foundation Trust	Tremona Road, Southampton, SO16 6YD
Dr	Mohan Shenoy	Consultant Paediatric nephrologist	Royal Manchester Children’s Hospital, Manchester University Hospitals NHS Trust,	Royal Manchester Children’s Hospital, Manchester University Hospitals NHS Trust, Oxford Rd Manchester M13 9WL UK
Dr	Vinay Shivamurthy	Consultant Paediatric Rheumatology	Evelina London Children’s Hospital	Westminster Bridge Road London SE1 7EH
Ms	Theresa Simangan	Senior Paediatric Research Nurse	Whipps Cross University Hospital, London	Whipps Cross Road, Leytonstone, London, E11 1NR
Dr	Jaspal Singh	Paediatric Neurology Consultant	University Hospital Southampton NHS Foundation Trust	Tremona Road, Southampton, SO16 6YD
Mrs	Samantha Small	Paediatric Rheumatology Clinical Nurse Specialist	University Hospital Southampton NHS Foundation Trust	Tremona Road, Southampton SO16 6YD
Dr	Ameenat Lola Solebo	Consultant Paediatric Ophthalmology	Great Ormond Street Hospital for Children NHS Foundation Trust, Population, Policy & Practice Research & Teaching Department, University College London Great Ormond Street Institute of Child Health	Great Ormond Street, London WC1N 3JH, 30 Guilford Street, London WC1N 1EH
Dr	Helen Spencer	Consultant in Transplant and Respiratory Medicine	Great Ormond Street Hospital for Children NHS Foundation Trust	Great Ormond Street, London WC1N 3JH
Dr	Isaac Staff	Foundation Doctor	James Paget University Hospital	Lowestoft Rd, Gorleston, NR31 6LA
Dr	Karnam Sugumar	Consultant Paediatrician	Department of Child Health, Lancashire Teaching Hospitals NHS Trusts	Royal Preston Hospital, Sharoe Green Lane, Preston PR2 9HT
Ms	Zoe Swash	Clinical research coordinator	NIHR Southampton Clinical Research Facility, University Hospital Southampton NHS Foundation Trust	Tremona Road, Southampton, SO16 6YD
Dr	Sneha Tandon	Consultant Paediatric Haematologist	University Hospital Southampton NHS Foundation Trust	Tremona Road, Southampton SO16 6YD
Dr	Alexander W Tarr	Assistant Professor in Molecular Virology	W/A1328 Queen’s Medical Centre, School of Life Sciences	The University of Nottingham, NG7 2UH
Dr	Marc Tebruegge	Consultant in infectious diseases and immunology	Evelina London Children’s Hospital	Westminster Bridge Road London SE1 7EH
Ms	Evelyn Thomson	Research Nurse	Great North Children's Hospital	Royal Victoria Infirmary, Newcastle upon Tyne, NE1 4LP
Dr	Mark Tighe	Consultant Paediatrician	Poole Hospital NHS Foundation Trust	Longfleet Rd, Poole, BH15 2JB
Prof	Paddy Tighe	Assistant Professor	School of Life Sciences, University of Nottingham	Nottingham, NG7 2RD
Mrs	Joanne Tomlinson	Research Nurse	University Hospitals of North Midlands NHS Trust	Staffordshire Children’s Hospital at Royal Stoke, Newcastle Road, Stoke on Trent, ST4 6QG
Dr	Nicola Trevelyan	Consultant Paediatric Diabetologist	University Hospital Southampton NHS Foundation Trust	Tremona Road, Southampton, SO16 6YD
Dr	Brigitte Vollmer	Paediatric Neurology Consultant	University Hospital Southampton NHS Foundation Trust	Tremona Road, Southampton, SO16 6YD
Dr	Woolf Walker	Paediatric Respiratory Consultant	PCD Centre, University Hospital Southampton NHS Foundation Trust, School of Clinical and Experimental Sciences, Faculty of Medicine and Institute for Life Sciences, University of Southampton, NIHR Southampton Biomedical Research Centre, University Hospital Southampton NHS Foundation Trust	Tremona Road, Southampton SO16 6YD
Dr	Jo Walsh	Consultant Paediatric Rheumatology	Royal Hospital for Children Glasgow	345 Govan Rd, Glasgow G51 4TF
Ms	Rachel Wane	Lead Research Nurse – Children’s Team	Bradford Teaching Hospitals NHS Foundation Trust	Duckworth Lane, Bradford, West Yorkshire, BD9 6RJ
Dr	Evangeline Wassmer	Paediatric Neurology Consultant	Neurology Dept, Birmingham Children’s Hospital, Birmingham Women's and Children's NHS Foundation Trust	Steelhouse Lane, Birmingham B4 6NH

Mrs	Elizabeth Waxman	Paediatric Research Nurse Manager	Glasgow Clinical Research Facility	345 Govan Rd, Glasgow G51 4TF
Prof	Lucy R Wedderburn	Consultant Paediatric Rheumatology	Infection, Immunity and Inflammation Research & Teaching Department, University College London Great Ormond Street Institute of Child Health, Paediatric Rheumatology, Great Ormond Street Hospital NHS Foundation Trust, NIHR Great Ormond Street Hospital Biomedical Research Centre, Arthritis Research UK Centre for Adolescent Rheumatology, GOS Institute of Child Health, University College London	30 Guilford Street, London, WC1N 1EH, Great Ormond Street, London WC1N 3JH
Mrs	Lucy Wellings	Paediatric and Adolescent Research Nurse	University College London NHS Foundation Trust	3rd Floor Central, 250 Euston Road, London, NW1 2PG
Dr	Andrea Whitney	Paediatric Neurology Consultant	University Hospital Southampton NHS Foundation Trust	Tremona Road, Southampton, SO16 6YD
Dr	Elizabeth Whittaker	Consultant Paediatric Infectious Diseases	Imperial College Healthcare NHS Trust and Imperial College London. Children’s Clinical Research Facility	2nd Floor Cambridge Wing, Norfolk Place, London W2 1NY
Mrs	Rachel Wiffen	Paediatric Research Practitioner	Nottingham Children’s Hospital, Queen’s Medical Centre, Nottingham University Hospitals NHS Trust	Derby Road, Nottingham, NG72UH
Mr	Matthew Wilkins	Clinical research coordinator	NIHR Southampton Clinical Research Facility, University Hospital Southampton NHS Foundation Trust	Tremona Road, Southampton SO16 6YD
Ms	Jessica Williams	Data Manager	Great North Children's Hospital	Royal Victoria Infirmary, Newcastle upon Tyne, NE1 4LP
Dr	Mark Wood	Consultant Paediatric Rheumatology	Leeds Children Hospital	Leeds Teaching Hospital NHS Trust, Great George Street, LS1 3EX
Mrs	Sophie Wool	Research Nurse	Paediatric Oncology and Haematology, Cambridge University Hospitals NHS Foundation Trust	Hills Road, Cambridge, CB2 0QQ
Ms	Suzannah Wright	Project Manager	Paediatric Infectious Diseases Research Group, St George's, University of London	Cranmer Terrace, London, SW17 0RE
Mrs	Wing Han Wu	Clinical Research Coordinator	Centre for Adolescent Rheumatology Versus Arthritis, University College London	3rd Floor Central, 250 Euston Road, London, NW1 2PG
Ms	Caroline Youle	Respiratory Nurse Specialist/ Research Nurse	Nottingham Children’s Hospital, Queen’s Medical Centre, Nottingham University Hospitals NHS Trust	Derby Road, Nottingham, NG72UH
